# Springfield Healthy Hearts Data Framework: Protocol for a Co-Designed, City-Wide, Multicomponent Initiative That Coordinates, Monitors, and Evaluates Place-Based Heart Health Action

**DOI:** 10.2196/92825

**Published:** 2026-07-22

**Authors:** Lauren Ball, Kirsten N Adlard, David Chua, Amy Kirkegaard, Genevieve N Healy, Sarah A McNaughton, Jason Ferris, Sonia Shah, George Thomas, Benjamin J Seligmann, Anne Bernard

**Affiliations:** 1 Faculty of Health, Medicine and Behavioural Sciences, Centre for Community Health and Wellbeing, The University of Queensland, Springfield Tower, Level 7, 145 Sinnathamby Blvd, Springfield Central, Queensland, 4300, Australia, 61 413031470; 2 Faculty of Health, Medicine and Behavioural Sciences, School of Human Movement and Nutrition Sciences, The University of Queensland, St Lucia, Queensland, Australia; 3 National Heart Foundation of Australia, Brisbane, Queensland, Australia; 4 Faculty of Health, Medicine and Behavioural Sciences, Health and Wellbeing Centre for Research Innovation, The University of Queensland, St Lucia, Queensland, Australia; 5 Faculty of Health, School of Exercise and Nutrition Sciences, Deakin University, Geelong, Victoria, Australia; 6 Faculty of Health, Medicine and Behavioural Sciences, Queensland Digital Health Centre, The University of Queensland, Herston, Queensland, Australia; 7 School of Public Health, Faculty of Health, Medicine and Behavioural Sciences, The University of Queensland, Herston, Queensland, Australia; 8 Institute for Molecular Bioscience, The University of Queensland, St Lucia, Queensland, Australia; 9 Australian Research Council Centre of Excellence for the Digital Child, Kelvin Grove, Brisbane, Queensland, Australia; 10 Faculty of Engineering, Architecture and Information Technology, Sustainable Minerals Institute, The University of Queensland, St Lucia, Queensland, Australia; 11 UQ Data Science Collaborative Research Platform (UQ DS CRP), The University of Queensland, St Lucia, Queensland, Australia

**Keywords:** cardiovascular diseases, public health, cross-sectional studies, community-based participatory research, learning health system, primary prevention

## Abstract

**Background:**

Cardiovascular disease remains the leading global cause of mortality, driven by interrelated behavioral, biological, and psychosocial risk factors, despite the availability of effective prevention and treatment strategies. Persistent policy inertia, systemic fragmentation, and adverse social and commercial determinants have limited national responses. Addressing these gaps necessitates place-based, systems-oriented approaches that mobilize local assets, engage multisector stakeholders, and incorporate adaptive evaluation. The *Springfield Healthy Hearts* initiative exemplifies such an approach by positioning Greater Springfield as a “living laboratory” for coordinated cardiovascular health action through a comprehensive data framework, providing a replicable model for other communities.

**Objective:**

This protocol outlines the *Springfield Healthy Hearts* Data Framework, a multicomponent system for dynamically guiding, implementing, and evaluating coordinated action for heart health.

**Methods:**

The data framework was developed through a structured co-design process involving community members, expert researchers, health professionals, and representatives from local implementation partners. The framework comprises four integrated components: (1) *Project evaluation*, applying pragmatic frameworks to assess coordinated action projects; (2) *Community evaluation*, a repeated cross-sectional evaluation of Springfield residents, workers, and regular visitors to capture individual-level behavioral, biological, and psychosocial cardiovascular disease risk factors, as well as engagement with coordinated action projects; (3) *City evaluation*, ongoing monitoring of suburb- and city-level indicators across 4 domains (sociodemographic characteristics, built environment, food and commercial environment, and health services); and (4) *Data synthesis*, to utilize data across all levels to inform a continuous learning system. Project evaluations will use both quantitative and qualitative methods, including realist evaluation where appropriate. Community evaluation will be analyzed using descriptive statistics, mixed effects models, and subgroup analyses, with missing data addressed via multiple imputation. City-level data will be analyzed descriptively and dynamically to detect temporal trends and contextual changes.

**Results:**

Initial funding for the *Springfield Healthy Hearts* Data Framework was secured in June 2025. Co-design workshops were conducted between November 2025 and February 2026 (n=15 participants), informing the design and prioritization of framework components. Community evaluation data collection is scheduled to commence in September 2026 and conclude in August 2027. Data cleaning and preliminary analyses are anticipated in late 2027, with first results expected to be disseminated in early 2028.

**Conclusions:**

The *Springfield Healthy Hearts* Data Framework is a replicable model for other communities aiming to implement city-wide, coordinated approaches to heart health action. Findings will be disseminated through peer-reviewed publications, community reports, interactive dashboards, and policy briefs.

## Introduction

### Background

Cardiovascular disease (CVD) remains the leading cause of mortality globally, accounting for an estimated 20.5 million deaths in 2021, a 60% increase since 1990 [1]. The burden is driven by a complex interplay of modifiable behavioral risk factors (eg, low diet quality, physical inactivity, alcohol, and tobacco use), biological risk factors (eg, hypertension, dyslipidemia, impaired glucose regulation), and psychosocial risk factors (eg, stress, depression, and social isolation) [2]. Despite the availability of cost-effective prevention, detection, and treatment strategies, national responses have been insufficient due to policy inertia, systemic fragmentation, and the negative influence of detrimental social and commercial determinants of health [3].

Addressing this knowledge-action gap requires place-based, systems-oriented initiatives that mobilize local assets, engage multisector stakeholders, and embed adaptive evaluation in real-world contexts [4,5]. Such initiatives can leverage systems science to identify and respond to dynamic changes over time [5], generate locally relevant evidence to guide action, and contribute to broader policy and practice [5]. However, few existing models have operationalized comprehensive, co-designed data frameworks to sustain coordinated action for cardiovascular health.

Greater Springfield, a rapidly growing master-planned city in South-East Queensland, Australia, provides a unique setting for such an initiative. Its defined geographic boundaries, integrated urban infrastructure, and strong local governance align to suggest its relevance as a “living lab,” a place where multistakeholder collaboration, embedded research, and iterative development can address complex health challenges. The *Springfield Healthy Hearts* initiative positions Greater Springfield as a demonstration city for heart health through coordinated, systems-based action. Our previous work described the conceptual foundations of *Springfield Healthy Hearts*, including its systems ontology, transdisciplinary epistemology, and methodological principles.

This paper will build upon the *Springfield Healthy Hearts* concept by outlining the *Springfield Healthy Hearts* Data Framework, a multicomponent system that will be implemented to guide, monitor, and evaluate coordinated action for heart health in Springfield. By specifying how we will translate the *Springfield Healthy Hearts* vision through the operation of the data framework, this protocol will provide a replicable model for other communities to follow.

### Aims and Objectives

#### Aim

The aim of this study is to establish a multicomponent data framework that can be used to inform and evaluate coordinated action for heart health in Springfield, Australia.

#### Objectives of the Data Framework

The objectives of the data framework are defined as follows:

1. *Project evaluation:* to evaluate the reach, fidelity, effectiveness, adoption, and sustainability of coordinated action projects that are undertaken as part of *Springfield Healthy Hearts*.
2. *Community evaluation:* to regularly evaluate a representative sample of the Springfield community, capturing individual-level behavioral, biological, and psychosocial risk factors for CVD, as well as engagement with coordinated action projects.
3. *City evaluation:* to monitor suburb-level and city-level features that indicate factors known to affect heart health outcomes.
4. *Data synthesis:* to develop a process for capturing, interpreting, and reporting data from the first 3 components of the framework, enabling a continuous learning system that guides ongoing coordinated action in Springfield.

## Methods

### Program Design and Approach

**Figure 1.**
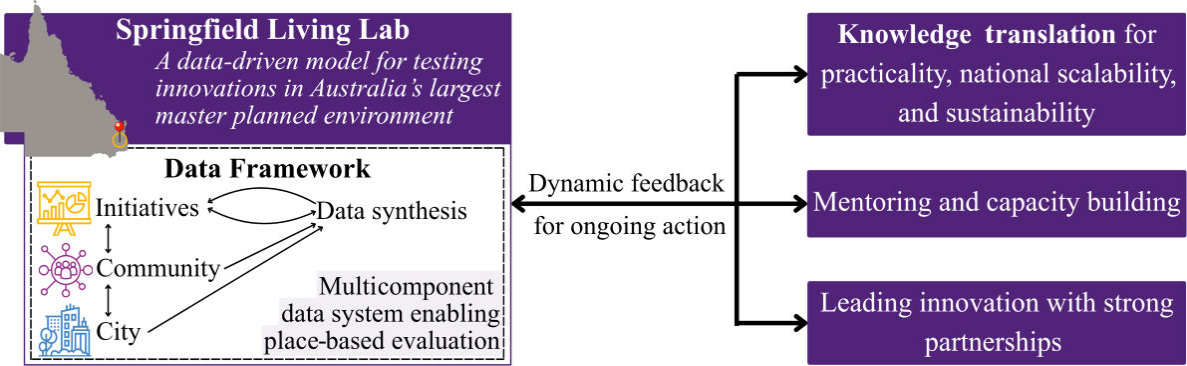
Overview of the *Springfield Healthy Hearts* Data Framework.

*Springfield Healthy Hearts* is grounded in a systems approach [6] that recognizes cardiovascular health as an emergent property (characteristic) of interconnected social, environmental, economic, and health care factors (Figure 1). The initiative’s ontology acknowledges that these systems are dynamic, adaptive, and shaped by interactions, ranging from individual behaviors to policy environments. The *Springfield Healthy Hearts* Data Framework operationalizes this approach using 3 interlinked components, followed by a fourth component that integrates and synthesizes the generated data. These components are designed to operate as an integrated system, with data flowing between levels to inform coordinated action.

Coordinated action projects will be quasiexperimental in design, with outcomes captured through the project evaluation component of the framework. Community-level risk for CVD will be assessed using a repeated cross-sectional evaluation through the community evaluation component of the framework. The city evaluation component will consist of a descriptive ecological design, collecting aggregate indicators known to affect heart health.

The data framework was developed through a structured, co-design process (University of Queensland Human Research Ethics Committee 2024/HE002099) involving community members, local health professionals, researchers, and local delivery partners and health services. The co-design process comprised iterative discussions with diverse informants to explore, refine, and prioritize research questions, data collection procedures, and outcome measures, ensuring local relevance and acceptability [7].

### Ethical Considerations

The data framework co-design process was approved by the University of Queensland Human Research Ethics Committee (2024/HE002099). Informed consent was obtained from participants prior to participation, and each participant received appropriate remuneration for their involvement as per Health Consumers Queensland guidelines [8]. Given the integration of multiple data sources, there is a potential risk of reidentification. This will be mitigated through deidentification procedures and secure data linkage protocols. Data will be analyzed and reported in aggregate form to protect participant confidentiality. AI analyses will be conducted on deidentified data and will not be used for individual-level decision-making. Future components mentioned in our protocol (eg, the coordination action projects) will undergo the appropriate research ethics committee review and approval process.

Adequate compensation (cash vouchers, approximately Aus $30 to $50 [Aus 1=US $0.69 as of July 8, 2026] per completed assessment component) for participants is essential to ensure participation and completion of the study and will be provided to participants after they complete each study component, in line with institutional ethical approval and intended to support participation without undue inducement. Participants will be given the option to “donate” their incentives back to the study if they wish. For participants less than 18 years of age, incentives will be provided to their parent/guardian.

### Components of the Springfield Healthy Hearts Data Framework

#### Component 1: Coordinated Action Project Evaluations

##### Overview

Coordinated action refers to “real-world” implementation projects that involve active contribution between more than one segment of the community (eg, health, social services, schools, retail, sporting teams, and community organizations) [9]. Groups and community members work together, supported by the project team, to jointly plan, implement, and monitor innovation or change toward a shared desired outcome, with exchanges of expertise, resources, and responsibilities. In this case, the shared desired outcome is the translation of evidence into fit-for-purpose action for heart health.

##### Identifying Potential Coordinated Action Projects

Prospective coordinated action projects will be identified through ongoing needs assessment activities, drawing on data sources to ensure projects address the highest-priority drivers of heart health in Springfield. Data sources include census data, national and regional health publications, and local informant engagement. As the data framework matures, prospective coordinated action projects will also be informed by the *Springfield Healthy Hearts* Data Framework. Prospective projects will be prioritized based on expected impact, feasibility, alignment with the *Springfield Healthy Hearts* vision, and potential to reduce inequities (Multimedia Appendix 1).

##### Endorsing Coordinated Action Projects

Prioritized projects will be reviewed and endorsed by the *Springfield Healthy Hearts* Alliance, ensuring they are supported by key community, health, academic, and industry partners. The Alliance comprises public and private organizations, not-for-profit entities, and community members with strong connections to the Springfield community. Guided by a community-developed set of values and principles that respect community ownership, unity, ethics, and sustainability, the Alliance serves as the overarching governing committee overseeing the progress of coordinated action projects and the broader data framework. This endorsement process will verify that each project aligns with the Alliance’s agreed-upon values and priorities, contributes to system-wide change, and has committed implementation partners.

##### Developing and Implementing Coordinated Action Projects

Each coordinated action project will progress through a structured development cycle, informed by community-based participatory research [10], implementation science [11], and health promotion theories [12]. The 11 steps comprise the following:

1. *Engage*—connect with local community members, partners, and service providers, as relevant, who are interested in the topic/priority/problem
2. *Inform*—review evidence on “problem” and integrate community insights
3. Prioritize—confirm objectives, target population, and settings based on an informed understanding of the “problem”
4. *Develop*—co-design project components, logic model, and evaluation plan
5. *Pilot*—test feasibility and acceptability on a small scale
6. *Evaluate*—assess reach, fidelity, adoption, and preliminary outcomes
7. *Implement*—scale up within Springfield as appropriate
8. *Optimize and integrate*—refine delivery and embed into routine practice
9. *Sustained deployment*—explore sustained delivery options, including commercial partnerships, to support broader roll-out
10. *Adopt*—promote the ongoing uptake by organizations and community groups
11. *Monitor impact*—link program exposure to cohort and city-level outcomes to estimate the contribution to population health improvement

##### Examples of Coordinated Action Projects

The following examples represent early priority coordinated action projects, co-designed with community stakeholders, which are currently underway and will contribute data to the *Springfield Healthy Hearts* Data Framework:

- *Heart health screening and referral pathways*—integration of nonclinical heart health screening and streamlined referral processes to general practice (presently at step 3).
- *Jump rope for heart*—long-term active engagement of Springfield schools in the Heart Foundation’s flagship program (presently at step 4).
- *Community cooking interventions*—skills-based education in Mediterranean diet principles and healthy cooking (presently at step 5).
- *Social prescribing for heart health*—nature-based physical activity program for heart health via informal referrals from health providers (presently at step 5).

The RE-AIM (reach, effectiveness, adoption, implementation, maintenance) and PRISM (perspectives, characteristics of implementing settings, external environment, implementation and sustainability infrastructure) frameworks will be used to guide both the design and evaluation of coordinated action projects within the *Springfield Healthy Hearts* Living Lab. In this context, these frameworks are adapted to reflect the multisector and place-based nature of the interventions. For example, Reach is operationalized at both individual and community levels, including participation across diverse population groups. Effectiveness includes both project aims, health outcomes, and broader indicators of well-being as appropriate to each project. Adoption and implementation are considered across partner organizations, recognizing variation in capacity and context. Maintenance is conceptualized not only at the individual level but also in terms of the sustainability of coordinated action. PRISM will be used to account for contextual factors, including organizational characteristics, community environment, and policy influences, which may affect implementation and outcomes. These adaptations enable the frameworks to be applied to coordinated, real-world interventions operating across multiple sectors. The RE-AIM-PRISM [13] reporting frameworks will ensure that data are pragmatic and contextually relevant, informing continuous improvement and sustainability across topics, populations, and settings.

##### Evaluation of Coordinated Action Projects

Data from coordinated action projects will be analyzed using descriptive statistics and, where appropriate, regression models to assess reach, engagement, and short-term outcomes. Analyses will be tailored to the design of each project and may include pre-post comparisons or subgroup analyses. We will evaluate the reach, fidelity, effectiveness, adoption, and sustainability of all coordinated action projects undertaken as part of *Springfield Healthy Hearts* (Table 1). Each project will follow a standardized evaluation protocol [14], drawing on delivery reports, administrative records, surveys, interviews, and focus groups, as relevant. Analyses will employ descriptive, quantitative, and qualitative approaches, including realist evaluations [15]. A dynamic database (already prototyped) will catalog all coordinated action projects, linking objectives, target populations, and delivery settings. Where feasible and with appropriate participant consent, we will link intervention exposure to community-level and city-level outcomes using data linkage to estimate the contribution to observed changes.

**Table 1.** Template for evaluating coordinated action projects within *Springfield Healthy Hearts*.

Domain	Outcome measure	Data sources
Project overview	Project title Lead organizations and partners involved Theory of Change Demographics and inclusion/exclusion criteria	Project proposal and protocol
Settings and locations	Geographic area(s) within Springfield Setting type (eg, school, workplace, and community center) and related physical and personnel infrastructure	Project proposal and protocol
Reach	Number of people reached, eligible, engaged, and completed, as relevant Demographic characteristics of participants Strategies used to enhance equity of participation	Registration lists, postproject survey, and implementation team reflections
Fidelity and adaptations	Core components delivered as intended Adaptations made during delivery	Postproject survey and implementation team focus group
Adoption	Organizations/sites approached, engaged, adopted, sustained, and sustained Reasons for adoption or nonadoption	Administrative logs, key informant interviews, and site audits
Effectiveness	Changes in effectiveness outcomes (eg, behaviors, knowledge, and health indicators) Unintended consequences	Pre/postproject surveys
Implementation	Acceptability, appropriateness, and feasibility, including resources used (time, staff, and costs) Barriers and facilitators to implementation	Delivery records, implementation team survey, or focus group
Sustainability and scalability	Continuation plans beyond initial funding Potential for scale-up or replication	Postproject survey, process outcome interviews, and implementation team reflections
Synthesized learnings	Key insights for future projects Recommendations for system-level action	Process outcome interviews and implementation team reflections

### Component 2: Community Evaluation

#### Overview

We will conduct a cross-sectional evaluation of heart health risk factors in the Springfield community, with repeat waves every 3 years to monitor changes over time. This approach and timeline enable ongoing surveillance of the Springfield community, representative of key sociodemographic characteristics, while minimizing participant burden and resource demands associated with cohort retention. It is also consistent with leading population health monitoring systems such as the US National Health and Nutrition Examination Survey, which uses repeated cross-sectional sampling to generate nationally representative data on health and risk factors [16].

#### Potential Sample

Springfield residents of all ages, sexes, ethnicities, cultural heritages, and socioeconomic status, with additional consideration given to people who work in or regularly engage with Springfield (defined as >4 visits lasting >1 h each per month).

#### Sampling Strategy

To maximize participation and diversity, we will use probability sampling at the household level, enabling enrollment of multiple members per home and capturing a wide range of ages. There will be a deliberate emphasis on including children and adolescents, as well as genetically related participants, to allow exploration of familial clustering of CVD risk [17]. Recruitment will be stratified using census-derived indicators of cultural diversity and socioeconomic status, ensuring the adequate representation of priority populations and enabling subgroup analyses to address health equity questions. While probability-based sampling strategies are employed, the potential for sampling and nonresponse bias remains, particularly if participation differs across sociodemographic groups. To assess this, characteristics of the study sample will be compared with census population data. Where appropriate, survey weighting and stratified analyses will be used to improve representativeness and account for differential participation. Participation rates will also be monitored throughout data collection to identify and respond to any emerging biases.

#### Target Sample Size

Based on an eligible population of 56,000 individuals, a target sample size of 1040 people per wave was determined using a 95% CI and ±3% precision (*P*=.05 and Z=1.96). Allowing for a 50% response rate, we will invite approximately 2100 residents to achieve the required completion [18,19].

#### Recruitment

Recruitment will be embedded within a city-wide heart health campaign, designed to maximize visibility and community engagement across Springfield. This campaign will use multiple channels, including social media, local newspapers, radio, schools, workplaces, sporting clubs, and community gathering spaces. Materials will be culturally tailored, translated into languages commonly spoken in the region, and presented in accessible formats to maximize inclusivity. The campaign will showcase opportunities for local preexisting education, events, and participation in coordinated action projects, with an invitation to participate in the community evaluation. This integrated approach is intended to normalize participation as a civic contribution to Springfield’s health future. Recruitment will continue pragmatically until the desired sample size is reached. Interested individuals will be directed to a dedicated website and secure digital portal that enables self-registration. A trained research staff member will provide in-person and virtual enrollment support until the target is completed (expected within 12 mo).

#### Dynamic, Tiered Consent

We will utilize a dynamic, tiered consent process that enables participants to select the level of involvement most suitable for them and to modify their consent over time. Beyond the outcomes described below (Table 2), permission for data linkage will be sought, and information about coordinated action projects will be disseminated. Youth assent and parent/guardian legal consent will be sought for participants aged below 16 years [20]. This adaptive approach will maximize recruitment, respect participant autonomy, and sustain engagement in other projects within *Springfield Healthy Hearts*. Participants who were less than 16 years old and consented in previous data collection rounds and who want to participate in an additional data collection round after they reach 18 years will be reconsented and assigned a unique participant identifier [21,22]. Participants will be able to adjust these preferences over time, with support from the digital portal, the ability to contact the research team at any time, and periodic communication.

**Table 2.** Core outcome measures for the *Springfield Healthy Hearts* community evaluation.

Domain and outcome measure	Instrument/tool
Behavioral	
Diet quality	Food Frequency Questionnaire assessed using the Dietary Guideline Index [23]
Physical activity/sitting time	International Physical Activity Questionnaire—Short Form (IPAQ-SF) [24], supplemented by the frequency of resistance training and daily step count in a subsample with a linked smartwatch [25]
Smoking and vaping	Self-report using validated approach (current, former, never, frequency, and intensity) [26]
Alcohol	Alcohol Use Disorders Identification Test—Consumption (AUDIT-C) [27]
Sleep health	Sleep duration and sleep quality via RU SATED^a^ [28]
Screen time	Movement Behaviour Questionnaire [29]
Biological	
Anthropometry	Height using stadiometer; weight using calibrated digital scales, waist circumference using fabric tape
Blood pressure	Digital sphygmomanometer, following WHO^b^ STEPS protocol (seated, 3 measures, average of last 2)
Blood glucose	Fasting blood glucose and HbA_1c_^c^ (venous blood sample)
Blood cholesterol	Fasting blood test (total cholesterol, HDL^d^, LDL^e^, and triglycerides), including lipoprotein(a) [30]
Physical capability	Hand grip strength
Psychosocial	
Anxiety	Kessler Psychological Distress Scale (K10) [31]
Depression	Depression, Anxiety and Stress Scale (DASS-21) [32]
Loneliness	UCLA-4^f^ item score [33]
Quality of life	WHO-5 Well-being Index [34]
Socio demographic characteristics and health status	
Age, sex, and gender	Self-report, aligned with ABS^g^ standard demographic questions [35]
Ancestry	ABS standard items and language spoken at home
Household composition	Self-report: dependents and caring roles [36]
Education and employment	ABS standard items; highest level of education, current employment status, and type [37]
Socioeconomic status	Index of Relative Socio-economic Advantage and Disadvantage (IRSAD) [38] via residential postcode plus self-reported income adequacy and housing tenure
Family history	Family history of premature CVD^h^ (coronary heart disease, cerebrovascular, peripheral vascular diseases, or stroke in the first degree relative >65 female relative, >55 male relative)
Health conditions (eg, type 2 diabetes, dementia, asthma, arthritis, and cancer)	ABS standard items, Self-report checklist of physician diagnoses; Self-report of regular medications
Disability status	ABS Short Disability Module [39]
Living lab inputs	
Coordinated action projects	Awareness and participation in coordinated action projects

a RU SATED: Regularity, Satisfaction, Alertness, Timing, Efficiency, and Duration. This is a 6-item sleep health scale to measure sleep duration and sleep quality.

b WHO: World Health Organization.

c HbA_1c_: glycated hemoglobin.

d HDL: high-density lipoprotein.

e LDL: low-density lipoprotein.

f UCLA-4: University of California Los Angeles Loneliness Scale–4 item.

g ABS: Australian Bureau of Statistics.

h CVD: cardiovascular disease.

#### Outcome Measures

The community evaluation will capture core behavioral, biological, and psychosocial risk factors for CVD, supported by community co-design (Table 2). As the study evolves, measures may expand through linkages to routinely collected datasets, maintaining a scalable and cost-efficient monitoring system.

#### Data Collection

Data will be collected using self-administered surveys and clinical assessments. Participants will complete surveys on tablets while providing biological measures (blood pressure, anthropometry, and venous blood) at the Springfield community research office in private data collection rooms. Where consent is provided, routine health measures may also be captured through general practice records, subject to linkage agreements. All resulting data will be stored within the University of Queensland Trusted Research Environment, using established infrastructure security controls. Robust data governance will be implemented to ensure that project partners do not receive copies of raw data. Instead, data visits will occur, allowing approved users to analyze the data within the secure environment without data leaving the University of Queensland’s computer systems. This approach provides strong protection for privacy, confidentiality, and long-term stewardship of participant information.

#### Planned Analyses

We will quantify and model behavioral, biological, and psychosocial risk factors across age, cultural, and socioeconomic groups within the Springfield community. Community-level data will be analyzed using generalized linear mixed models to account for clustering at the household level. Key covariates will include age, sex, socioeconomic position, and relevant behavioral and biological risk factors. Where appropriate, survey weighting will be applied to improve representativeness. Missing data will be addressed using multiple imputation, with sensitivity analyses conducted to assess robustness. Subgroup and interaction analyses (eg, by age group, sex, and contextual factors) will be performed within the mixed-model framework to identify differential risk patterns and to provide a baseline for data synthesis and future waves.

### Component 3: City Evaluation

#### Overview

The evaluation of Springfield at the city level will focus on environmental, social, and system-level factors known to influence heart health, along with proxy indicators of community behaviors and treatment patterns. Together, these data will form a comprehensive understanding of Springfield’s cardiovascular health environment, complementing the community and project evaluations. Data collection was pilot tested between 2023 and 2024 and will repeat every 3 years to monitor changes over time (Table 3):

1. *Administrative datasets*—analyses of publicly available and partner-shared datasets to describe demographic composition, socioeconomic status, health outcomes, and service utilization.
2. *Wastewater analysis*—quarterly sampling from key sanitary sewer trunk lines sampling points to provide proxy measures of population-level exposures and behaviors, including cardiovascular medications, diet, and mental health indicators.
3. *Spatial mapping*—integration of geographic information systems data, publicly available government records, and business directories to characterize the built environment, health services, and food environments.
4. *Field audits*—underpinned by citizen science methods, to capture local features such as streetscape quality, cycling infrastructure, shade, and in-store food environments.

Citizen scientists involved in field audits will receive standardized training, including written protocols, examples of completed assessments, supervised pilot activities where feasible, and have access to annual refresher and on-demand training. Data collection tools will be designed to ensure consistency, including structured checklists and clear variable definitions. To support data quality, a subset of audits will be repeated by trained research staff to assess interrater reliability. Ongoing quality control procedures will include periodic review of submitted data, identification of outliers or inconsistencies, and where appropriate, feedback to participants.

**Table 3.** City evaluation measures for *Springfield Healthy Hearts,* assessing people and place.

Domain	Outcome measure	Instrument/tool
People		
Population sociodemographic characteristics	Population size, age, cultural diversity, education, employment, and housing	ABS^a^ Census (public release, 5-yearly) and ABS Estimated Resident Population (annual, public release)
Population health characteristics	CVD^b^ incidence, mortality, hospitalizations, and emergency presentations	Queensland Health and Mater Hospital datasets
Wastewater surveillance	Indicators of medication use, smoking, diet (eg, vegetable metabolites and artificial sweeteners), and antidepressant use	Queensland Alliance for Environmental Health Sciences (QAEHS) laboratory
Place		
Food environment	Outlet density and type (supermarkets, greengrocers, fast food, and cafés) In-store product availability, pricing, and promotions	ABS Business Counts (public release) cross-checked with Google Places API (public access) Nutrition Environment Measures Survey in Stores (NEMS-S) audits and Healthy Diets ASAP^c^ tool
Health services environment	GP^d^ clinics, allied health, hospitals, and pharmacies	Healthdirect National Health Services Directory (public access), as well as ABS Business Counts (public release) cross-checked with Google Places API (public access)
Green space and recreation environment	Parks, ovals, gyms, and sports clubs	Queensland Land Use Mapping Program (QLUMP) (public release via Queensland Spatial Catalogue [QSpatial] dataset
Walkability, cycle infrastructure, and streetscape	Street connectivity, cycle safety, traffic safety, pedestrian infrastructure, and shade	Walkability via NEWS-AU^e^, cycle infrastructure via Bicycle Environment and Safety Audit (BESA), and Shade Audit Tool (Cancer Council Australia) conducted via research assistant-led field audits

a ABS: Australian Bureau of Statistics.

b CVD: cardiovascular disease.

c ASAP: Australian Standardised Affordability and Pricing.

d GP: general practitioner.

e NEWS-AU: Neighbourhood Environment Walkability Survey, Australian version.

#### Planned Analyses

City-level indicators will be analyzed descriptively, geospatially, and longitudinally using established analytic pipelines. Administrative and wastewater datasets will provide annual and high-frequency inputs on service utilization and proxy behaviors while mapping, and audits will update Springfield’s built and service environments on 3- to 5-year cycles. Analyses will employ descriptive statistics, benchmarking against Greater Ipswich and Queensland averages, and geospatial accessibility modeling (eg, proximity to health services, green space, and food outlets). All indicators will be geocoded and securely linked with individual heart health data (Component 2), enabling multilevel modeling of how Springfield’s structural and behavioral environments shape heart health outcomes over time. These analyses will be used to identify trends over time and variation across locations. Where feasible, multilevel models will be used to explore relationships between city-level factors and individual-level outcomes.

### Component 4: Data Synthesis

These data streams will feed into a live analytics environment that iteratively develops and tests theories of impact, explaining how coordinated action projects and environmental change influence heart health. Data synthesis will involve the integration of project-, community-, and city-level data using both traditional statistical approaches and exploratory methods. Multilevel modeling and pattern recognition techniques may be used to identify relationships across data sources. The handling of missing data will be tailored to the type and source of data within each component of the framework. Analyses will combine AI-enabled pattern recognition with structured hypothesis testing and safeguards, grounded in current evidence on CVD pathophysiology. These may include supervised and unsupervised machine-learning techniques, such as clustering and predictive modeling. These methods will be used to support the identification of relationships between coordinated action, environmental factors, and cardiovascular risk, particularly where traditional modeling approaches may be limited by complexity or dimensionality. Findings will be interpreted in collaboration with domain experts to ensure validity and relevance for decision-making. To minimize potential bias, model performance will be examined across key demographic subgroups, and input variables will be assessed for sources of structural bias. AI methods will be used to complement, rather than replace, hypothesis-driven analyses within the framework. Findings will be visualized through dynamic dashboards that provide real-time feedback, guiding decisions about priority coordinated action projects. Dashboard outputs will also support transparent reporting to partners, funders, policymakers, and the community. By embedding both traditional and emerging AI-enabled analytics within a tested systems architecture, the *Springfield Healthy Hearts* Data Framework will operate as a continuous learning and decision-support system, able to adapt, optimize, and scale coordinated action projects on heart health.

### Integration of Data Framework Components

The *Springfield Healthy Hearts* Data Framework is designed as an integrated, multilevel system rather than a set of discrete components. The 4 components operate together to support continuous learning and adaptive implementation. Coordinated action projects (component 1) generate data on implementation, reach, and effectiveness at the intervention level. Where participant consent is provided, these data may be linked to individual-level data collected through the community evaluation (component 2), enabling the examination of exposure-response relationships and individual risk profiles. City evaluation data (component 3) provides contextual information on environmental, social, and service-level factors, including health service utilization, built environment characteristics, and population-level trends. These data allow for the interpretation of intervention outcomes within the broader system context. Data synthesis (component 4) integrates these multilevel datasets through iterative analytical processes. Findings are translated into actionable insights and shared with implementation partners and the *Springfield Healthy Hearts* Alliance. These insights inform the refinement of existing coordinated action projects and prioritization of new initiatives. This process operates as a continuous feedback loop, in which data inform action and action generates new data. Over time, this enables adaptive system improvement and supports the development of scalable, evidence-informed approaches to CVD prevention.

### Data Access

All components of the data framework will be synthesized through a secure, continuously updated data management platform built on a secure Trusted Research Environment (TRE) infrastructure. The TRE ensures compliance with institutional, national, and international standards for data security and integrity, including encryption, role-based access, and audit trails. Data will be stored for a minimum of 15 years in line with the university’s research data management policy.

External researchers may apply for access to data through a formal application process. Applications will be reviewed by the *Springfield Healthy Hearts* Data Committee, which will assess proposals based on scientific merit, alignment with project objectives, ethical considerations, and data minimization principles. Approved researchers will access data within the TRE, where data remain deidentified and cannot be downloaded or removed. Access will be role-based and time-limited, with audit logs maintained to monitor data use. All external collaborations will require appropriate data sharing agreements and ethics approvals where applicable.

### Governance

The *Springfield Healthy Hearts* Alliance will meet quarterly and serve as the overarching governance body, bringing together research, community, and partner perspectives. The Alliance will be supported by the following three committees:

1. *Data committee*—responsible for data management, access, and linkage feasibility
2. *Community committee*—providing input on community priorities, data use acceptability, and ensuring decisions reflect Springfield’s values and lived experiences
3. *Knowledge translation and impact committee*—guiding the reporting and dissemination of findings, ensuring that outputs are relevant to stakeholders and inform ongoing coordinated action

The committees will meet quarterly and report to the Alliance, ensuring transparency, accountability, and alignment of data use with the strategic and ethical objectives of *Springfield Healthy Hearts*. This governance structure reflects our commitment to embedding community voice and translational pathways at the heart of the project.

Access to raw data will be restricted to the core *Springfield Healthy Hearts* research team, with permissions tailored according to roles and project responsibilities. Curated datasets may be accessed for approved analyses following a review by the project’s Data Committee. The Data Committee will oversee data governance, including the management of access requests, feasibility, and management of data linkages. External researchers may apply for data access through the committee, subject to data use agreements.

## Results

Funding for the *Springfield Healthy Hearts* Data Framework was secured in June 2025. Co-design workshops were conducted between November 2025 and February 2026 (n=15 community participants), informing the design and prioritization of framework components. Community evaluation data collection is scheduled to commence in September 2026 and conclude in August 2027. Data cleaning and preliminary analyses are anticipated in late 2027, with first results expected to be disseminated in early 2028.

## Discussion

### Overview and Contribution

This protocol describes a novel, multilevel data framework designed to support coordinated, adaptive approaches to CVD prevention at a city scale. By integrating data from coordinated action projects, community-level assessments, and city-level monitoring, the framework is expected to generate new insights into how behavioral, biological, and contextual factors interact in real-world settings. Importantly, this approach enables continuous learning, allowing interventions to be refined over time and supporting the development of scalable, evidence-informed strategies for improving cardiovascular health. This represents a shift from evaluating individual programs to understanding how prevention can be delivered effectively across an entire community.

### Strengths and Limitations

The *Springfield Healthy Hearts* Data Framework represents a novel, operationalized example of a place-based, co-designed, multilevel data system for cardiovascular health. Unlike traditional program evaluations or isolated surveillance activities, this framework integrates project-, community-, and city-level data within a continuous learning system, enabling real-time adaptation of coordinated action. By embedding systems thinking into design and implementation, *Springfield Healthy Hearts* aligns with global calls for interventions that recognize health as an emergent property of complex adaptive systems [40,41]. The co-design process ensures that the framework is grounded in local priorities and assets, enhancing its legitimacy and sustainability [7,42]. The integration of administrative, self-reported, and environmental data maximizes coverage and reduces blind spots in both population and system-level surveillance [43].

Several limitations should be acknowledged. First, some measures rely on self-reported data, which may be subject to recall or social desirability bias. Second, while data linkage across components offers analytical advantages, it introduces practical and methodological challenges, including ensuring data compatibility and maintaining data privacy. Third, the interpretation of city-level data carries a risk of ecological fallacy, whereby associations observed at the population level may not reflect individual-level relationships. These limitations are inherent to complex, real-world research and will be considered in the interpretation of findings.

Implementing a multilevel data framework in a real-world setting presents several challenges. These include coordinating across multiple sectors and organizations, maintaining consistent data quality across diverse data sources, and sustaining participant and partner engagement over time. To mitigate these challenges, the *Springfield Healthy Hearts* Living Lab adopts a collaborative governance model, with ongoing engagement of partners through the *Springfield Healthy Hearts* Alliance. Standardized protocols and training procedures are used to support data quality, while flexible implementation approaches allow adaptation to local contexts. Sustained engagement will be supported through regular communication, feedback of findings to partners and the community, and alignment of data activities with existing services and priorities. These strategies aim to enhance the feasibility, acceptability, and long-term sustainability of the data framework.

### Implications for Research and Practice

The *Springfield Healthy Hearts* Data Framework has implications for both research and practice in CVD prevention. For research, this framework demonstrates how multilevel data can be integrated to examine the dynamic relationships between individual risk factors, environmental conditions, and coordinated interventions in real-world settings. It provides a foundation for moving beyond the evaluation of isolated programs toward understanding how prevention operates as a system. For practice, the framework offers a structured approach for translating data into coordinated action across sectors, enabling health services, community organizations, and policymakers to respond more effectively to local needs. By supporting continuous learning and adaptation, this approach may improve the scalability and sustainability of prevention strategies. If successful, the framework will not only generate actionable insights for Springfield but will also provide a transferable model for other communities seeking to operationalize systems-based approaches to chronic disease prevention at scale.

Greater Springfield has several unique characteristics, including its master-planned design, rapid population growth, and integrated urban infrastructure, which may facilitate the implementation of coordinated, place-based approaches to health. These contextual factors may not be directly replicable in all settings. However, the underlying principles of the *Springfield Healthy Hearts* Data Framework, specifically the integration of multilevel data, coordinated action across sectors, and iterative learning within a real-world context, are likely to be transferable to other communities. Adaptation to the local context will be essential, and future work will explore how this framework can be applied in settings with different demographic, geographic, and organizational characteristics.

The sustainability of the *Springfield Healthy Hearts* Data Framework is supported by its integration into existing community, health service, and partner systems. The Living Lab approach facilitates alignment with local priorities and ongoing stakeholder engagement, which is critical for long-term implementation. While initial development is supported by research funding, future sustainability will be supported by a combination of continued research investment, partnerships with government and service providers, and, where feasible, integration of data activities into routine practice. This approach aims to embed the framework within the local system, rather than maintaining it as a standalone research initiative.

## Supplementary material

10.2196/92825 Multimedia Appendix 1 Springfield Healthy Hearts Vision.

## Abbreviations:

CVD: cardiovascular disease
GIS: geographic information systems
RE-AIM-PRISM: Reach, Effectiveness, Adoption, Implementation, Maintenance - Perspectives, Characteristics of Implementing Settings, External Environment, Implementation and Sustainability Infrastructure
TRE: Trusted research environment
