# Supplementary material for: Springfield Healthy Hearts Data Framework: Protocol for a Co-Designed, City-Wide, Multicomponent Initiative That Coordinates, Monitors, and Evaluates Place-Based Heart Health Action
Source: JMIR Res Protoc. 2026 Jul 22;15:e92825. doi: 10.2196/92825 (PMC13392651; doi:10.2196/92825)

Springfield Healthy Hearts

AT THE HEART

Springfield Healthy Hearts Vision

The core research foci of the Springfield Healthy Hearts vision:

Prevention

Supermarket choices, cooking interventions, physical activity programs,community awareness campaigns, Jump Rope for Heart, smoking cessation, plus more.

Early Detection, Diagnosis & Treatment

General practice screening tools, registries of people with cardiovascular disease, referral to specialists, social prescribing.

Support & Care

Cardiac rehabilitation, connected health professionals, best practice rehabilitation, discharge and recall, allied health models of care for after discharge.

Research & Evaluation

Longitudinal study of heart health at Springfield, wastewater monitoring of heart health indicators, and integration of AI.

The Springfield Living Lab concept and its path to global impact:

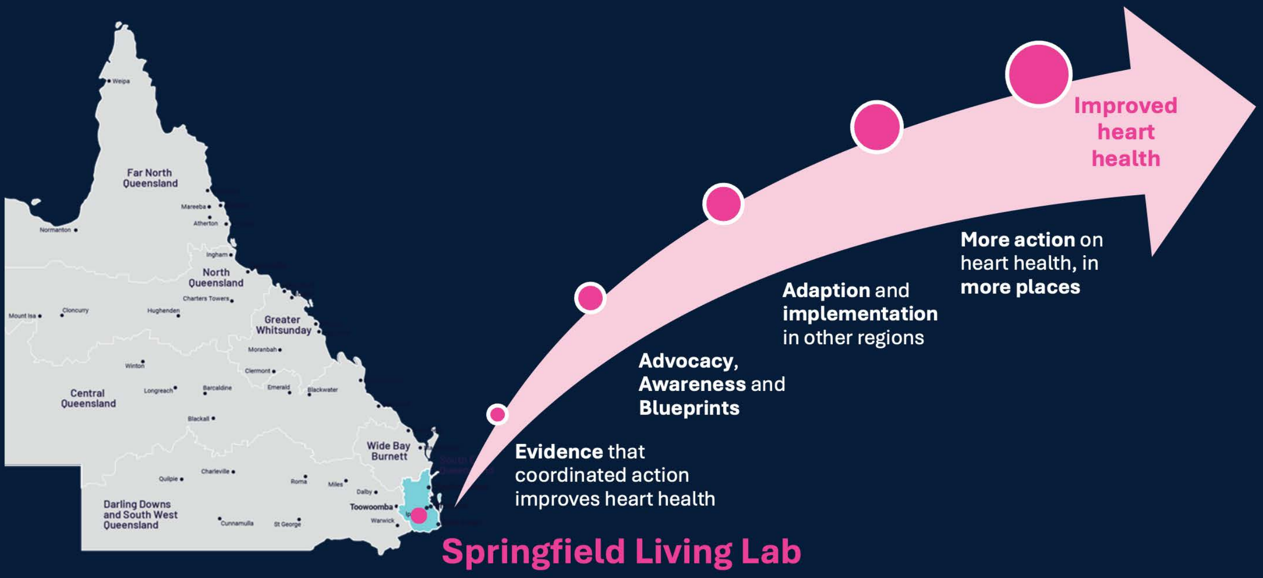

The data framework underpinning the Springfield Healthy Hearts vision:

A data-driven model for testing innovations in Australia's largest master-planned environment

Data Framework

Multi-component data system enabling place-based evaluation

Feedback Trident

Dynamic feedback for ongoing action

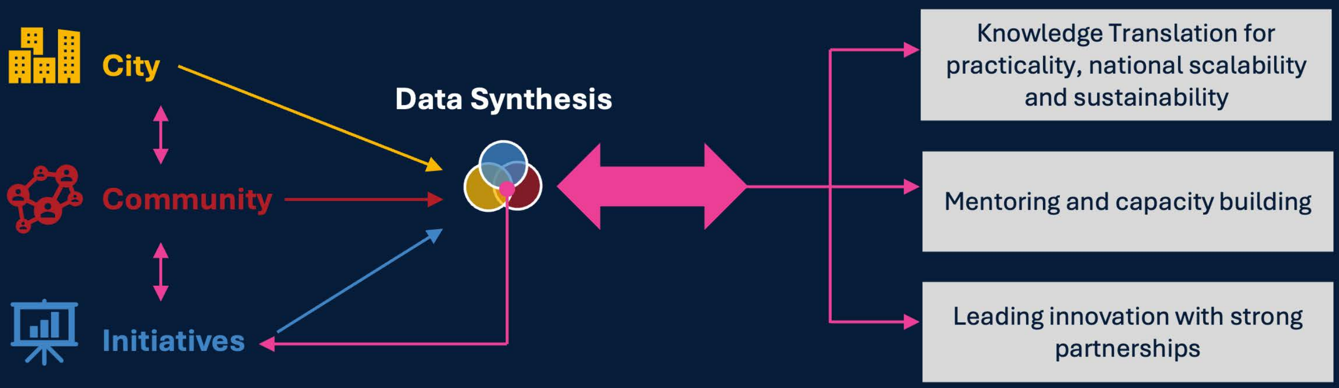

Supplement: Multimedia Appendix 1 [file resprot-v15-e92825-s001.pdf]
